# Vascular senescence and aging: mechanisms, clinical implications, and therapeutic prospects

**DOI:** 10.1007/s10522-025-10256-5

**Published:** 2025-05-26

**Authors:** Aitor Picos, Nuria Seoane, Manuel Campos-Toimil, Dolores Viña

**Affiliations:** 1 Physiology and Pharmacology of Chronic Diseases (FIFAEC), Center for Research in Molecular Medicine and Chronic Diseases (CiMUS), University of Santiago de Compostela, 15782 Santiago de Compostela, Spain; 2 Translational Research in Neurological Diseases (ITEN), Health Research Institute of Santiago de Compostela (IDIS), USC University Hospital Complex (CHUS), SERGAS, Santiago de Compostela, Spain; 3 Department of Pharmacology, Pharmacy and Pharmaceutical Technology, University of Santiago de Compostela, 15782 Santiago de Compostela, Spain

**Keywords:** Vascular senescence, Aging, DNA damage, Telomere attrition, SASP, ROS, Vascular pathologies, Therapeutic interventions

## Abstract

The aging vasculature is characterized by endothelial dysfunction, arterial stiffness, and increased susceptibility to vascular pathologies. Central to these changes is the process of cellular senescence, where endothelial and vascular smooth muscle cells lose their replicative and functional capacity and adopt a pro-inflammatory secretory phenotype. This review provides an overview of the key mechanisms underlying vascular senescence, including the p53/p21 and p16/Rb pathways, the senescence-associated secretory phenotype (SASP), and oxidative stress, examines its contribution to cardiovascular diseases in older adults, and highlights emerging therapeutic strategies aimed at delaying or reversing these age-related vascular changes. In vascular cells, DNA damage, oxidative stress, and chronic inflammation associated with aging converge to amplify senescence. Clinically, vascular senescence is linked with hypertension, atherosclerosis, and increased overall cardiovascular risk. Several interventions, ranging from senolytics to lifestyle factors, show promise in mitigating these changes; however, long-term studies are needed. Given that vascular senescence is a pivotal driver of cardiovascular pathology in aging, targeting senescent cells or their secretory phenotype may potentially offer new avenues for preventing or attenuating age-related vascular diseases. This review presents an updated and integrative overview of vascular senescence, connecting fundamental cellular mechanisms with their clinical manifestations and highlighting the most promising therapeutic interventions.

## Introduction

As life expectancy continues to rise worldwide, the burden of age-related diseases, particularly cardiovascular disorders, has become a major public health challenge (Niccoli and Partridge 2012). Cardiovascular diseases (CVDs) are the leading cause of death globally, accounting for nearly 18 million deaths annually, according to the World Health Organization (WHO). These diseases encompass a range of conditions, including coronary artery disease, stroke, heart failure, hypertensive heart disease, and arrhythmias (Roth et al. 2017). Among the various processes involved in cardiovascular aging, vascular senescence stands out as a central driver of endothelial dysfunction and arterial remodeling (Childs et al. 2015; Minamino et al. 2002).

Initially described as the “Hayflick limit” in fibroblasts by Hayflick and Moorhead (1961), senescence was originally characterized as a cellular process that permanently halts division once a critical replication threshold is reached (Hayflick 1964; Hayflick and Moorhead 1961). Proliferation cessation is accompanied by extensive morphological and phenotypical changes. Senescent cells often exhibit an enlarged and flattened morphology, increased granularity, and altered nuclear architecture, such as the formation of senescence-associated heterochromatin foci (SAHF) (Narita et al. 2003). These structural changes are complemented by metabolic reprogramming, including heightened lysosomal activity evidenced by increased beta-galactosidase levels (Dimri et al. 1995; Kurz et al. 2000; Lee et al. 2006). Furthermore, senescent cells actively secrete pro-inflammatory cytokines, growth factors, and proteases, collectively constituting the senescence-associated secretory phenotype (SASP) (Acosta et al. 2013; Coppé et al. 2008). These multifaceted changes highlight the complex roles of senescence in influencing both cellular functions and tissue homeostasis.

Senescence can be induced by a variety of mechanisms, including telomere shortening, oxidative stress, DNA damage, and oncogene activation. These triggers initiate complex signaling cascades, such as the p53/p21 and p16/Rb pathways, that lead to cell cycle arrest (d’Adda di Fagagna 2008; Sharpless and Sherr 2015). Moreover, the diversity of senescence phenotypes is evident in their context-dependent behavior: some senescent cells adopt a pro-inflammatory phenotype via the secretion of cytokines, growth factors, and proteases (Acosta et al. 2013; Birch and Gil 2020), while others exhibit altered metabolic activity or resistance to apoptosis (Tchkonia et al. 2013) or a combination of these traits (Giuliani et al. 2023).

Beyond their canonical roles in enforcing cell cycle arrest, the p53/p21 and p16/Rb pathways engage in intricate crosstalk with metabolic and inflammatory networks. For instance, p53 modulates mitochondrial respiration and glycolysis, linking DNA damage responses to cellular energy metabolism (Vousden and Ryan 2009). The mTOR pathway, often activated in aging, modulates senescence both directly and through SASP regulation and autophagy inhibition (Herranz and Gil 2018). Likewise, NF-κB, a key inflammatory regulator, is frequently activated in senescent cells, reinforcing SASP expression and contributing to a pro-inflammatory tissue microenvironment (Salminen et al. 2012). These interconnected signaling events shape the vascular senescence phenotype and provide potential therapeutic entry points.

Cellular senescence is one of the key hallmarks of aging, as proposed by Lopez-Otín et al. 2013, describing its central role in driving aging-related decline and pathologies. It serves as a crucial interface with other hallmarks, such as genomic instability and chronic inflammation, amplifying their effects. For instance, the accumulation of senescent cells exacerbates the pro-inflammatory milieu, which, in turn, accelerates tissue dysfunction and systemic aging (López-Otín et al. 2023; López-Otín et al. 2013). In the vascular context, senescence accelerates endothelial dysfunction and detrimental arterial remodeling, contributing to cardiovascular aging and diseases (Cheng et al. 2023; Grootaert et al. 2018; Minamino et al. 2002).

During aging, vascular senescence is characterized by the progressive accumulation of senescent endothelial and vascular smooth muscle cells within blood vessels (Childs et al. 2017; Gardner et al. 2015; Matthews et al. 2006). Senescent cells secrete pro-inflammatory cytokines, growth factors, and matrix-degrading enzymes as part of the SASP, exacerbating local and systemic inflammation. Over time, these changes impair endothelial function, reduce nitric oxide bioavailability, and promote arterial stiffening (Bloom et al. 2023; Gardner et al. 2015). This fosters a pro-thrombotic and pro-fibrotic environment, accelerating the development of cardiovascular diseases such as atherosclerosis and hypertension. The localized accumulation of senescent cells at sites of vascular injury highlights their direct role in disease progression, where they amplify tissue remodeling and contribute to plaque instability in atherosclerotic lesions (Bloom et al. 2022; Nagar et al. 2024; Sharpless and Sherr 2015).

**Fig. 1:**
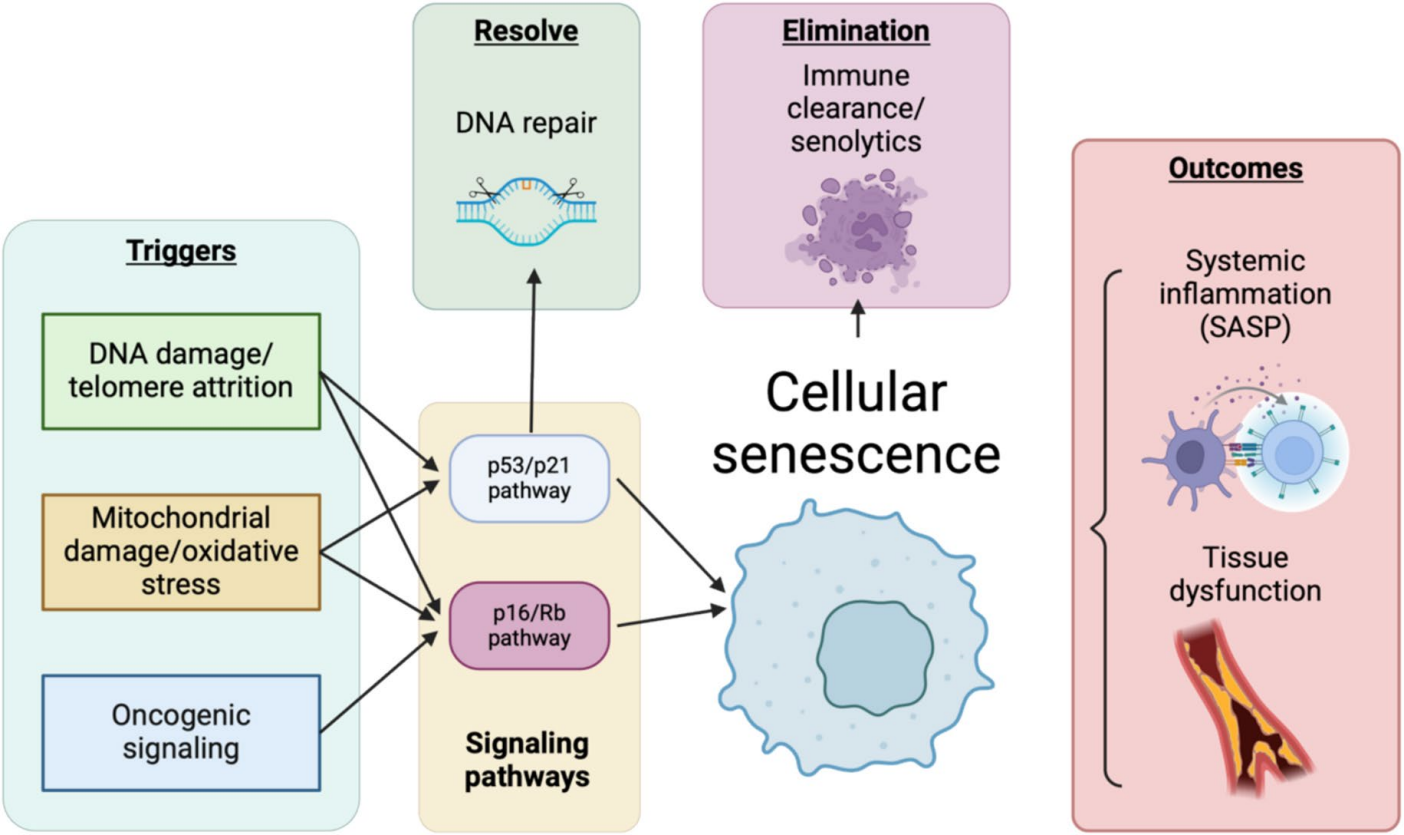
Overview on cellular senescence mechanism and modulation

Unraveling the mechanisms of vascular senescence offers critical insights into therapeutic targets that could slow or even reverse age-related cardiovascular decline (Fig. 1). For example, senolytic drugs, which selectively eliminate senescent cells, and senomorphic agents, which modulate the SASP, show promise in preclinical models (Aziz et al. 2024; Liu et al. 2024a; Liu et al. 2024b; Zhu et al. 2016; Zhu et al. 2015). However, challenges remain in translating these therapies into clinical applications, including potential off-target effects, delivery mechanisms, and long-term safety concerns. Additionally, interventions like caloric restriction mimetics and regular exercise have been demonstrated to mitigate vascular aging by reducing oxidative stress and enhancing endothelial function (Madeo et al. 2019; Seals et al. 2016).

Senescence can be triggered by various stressors, including DNA damage, telomere attrition, mitochondrial damage, oxidative stress, and oncogenic signaling. These triggers activate key signaling pathways, primarily the p53/p21 and p16/Rb pathways, leading to a stable cell cycle arrest. While senescence can prevent the proliferation of damaged cells, persistent senescent cells contribute to aging and disease through the secretion of a pro-inflammatory senescence-associated secretory phenotype (SASP), driving systemic inflammation and tissue dysfunction. Potential interventions, such as DNA repair mechanisms, senolytics/senomorphics, and immune clearance, aim to mitigate the detrimental effects of senescent cells. Created with BioRender.com.

Personalized medicine, supported by advancements in artificial intelligence (AI), holds immense potential in addressing age-related cardiovascular diseases (CVDs). AI algorithms can process vast and complex datasets, integrating genetic, epigenetic, and clinical data to uncover specific biomarkers linked to vascular aging and disease progression (Krittanawong et al. 2021; Topol 2019). The integration of personalized medicine with advanced AI technologies—ranging from large language models (LLM) to emerging AGI frameworks (Chakraborty et al. 2023; Mitchell 2024)—has the potential to revolutionize cardiovascular care, providing solutions that not only treat but also proactively prevent CVDs by targeting underlying mechanisms such as vascular senescence.

While significant progress has been made in understanding the individual mechanisms contributing to vascular senescence, there is a need for an updated and integrative overview that connects these fundamental cellular processes with their clinical manifestations and the most promising therapeutic interventions. This review aims to fill this gap by providing a comprehensive synthesis of the current knowledge on vascular senescence, bridging the gap between basic science and clinical applications in the context of cardiovascular aging.

## Mechanisms of vascular senescence

Vascular senescence is primarily induced by a combination of cellular stressors that accumulate with aging. Key inducers include telomere attrition, chronic oxidative stress, and persistent DNA damage, which trigger pathways such as the p53/p21 and p16/Rb signaling cascades to enforce cell cycle arrest (d’Adda di Fagagna 2008; Sharpless and Sherr 2015). Additionally, metabolic dysregulation, inflammation, and exposure to systemic risk factors like hypertension and hyperglycemia further accelerate senescence in vascular cells (Katsuumi et al. 2018; Minamino et al. 2002; Yamauchi et al. 2024). Beyond these drivers, the SASP exacerbates cellular dysfunction by reinforcing senescence within the tissue microenvironment. Concurrently, epigenetic modifications—spanning DNA methylation changes, histone modifications, and chromatin remodeling—are key in the whole senescence process. The following sections will explore in detail how these factors contribute to vascular senescence at the molecular and cellular levels.

### DNA damage and telomere attrition

Aging cells frequently accumulate DNA damage due to both external stressors, such as oxidative stress and ionizing radiation, and endogenous events like replication errors and metabolic byproducts. This accumulation often overwhelms cellular repair mechanisms, resulting in persistent DNA lesions that activate chronic DNA damage response (DDR) signaling (d’Adda di Fagagna 2008; Jackson and Bartek 2009). Telomere shortening with each cell division further compromises genome integrity. Once telomeres reach a critical length, protective shelterin complexes fail, exposing chromosome ends and triggering a cascade of DDR pathways (Cesare and Karlseder 2012; Ciccia and Elledge 2010; González-Amor et al. 2023).

DDR involves a complex network of signaling pathways that detect and repair DNA lesions. Key players include ataxia-telangiectasia mutated (ATM) and ataxia-telangiectasia and Rad3-related (ATR), which are protein kinases that phosphorylate downstream effectors such as H2AX, creating γ-H2AX foci. These foci serve as markers of DNA damage and recruit additional repair proteins to the damage sites, amplifying the response to genomic instability (Ciccia and Elledge 2010; Shiloh and Ziv 2013).

**Fig. 2:**
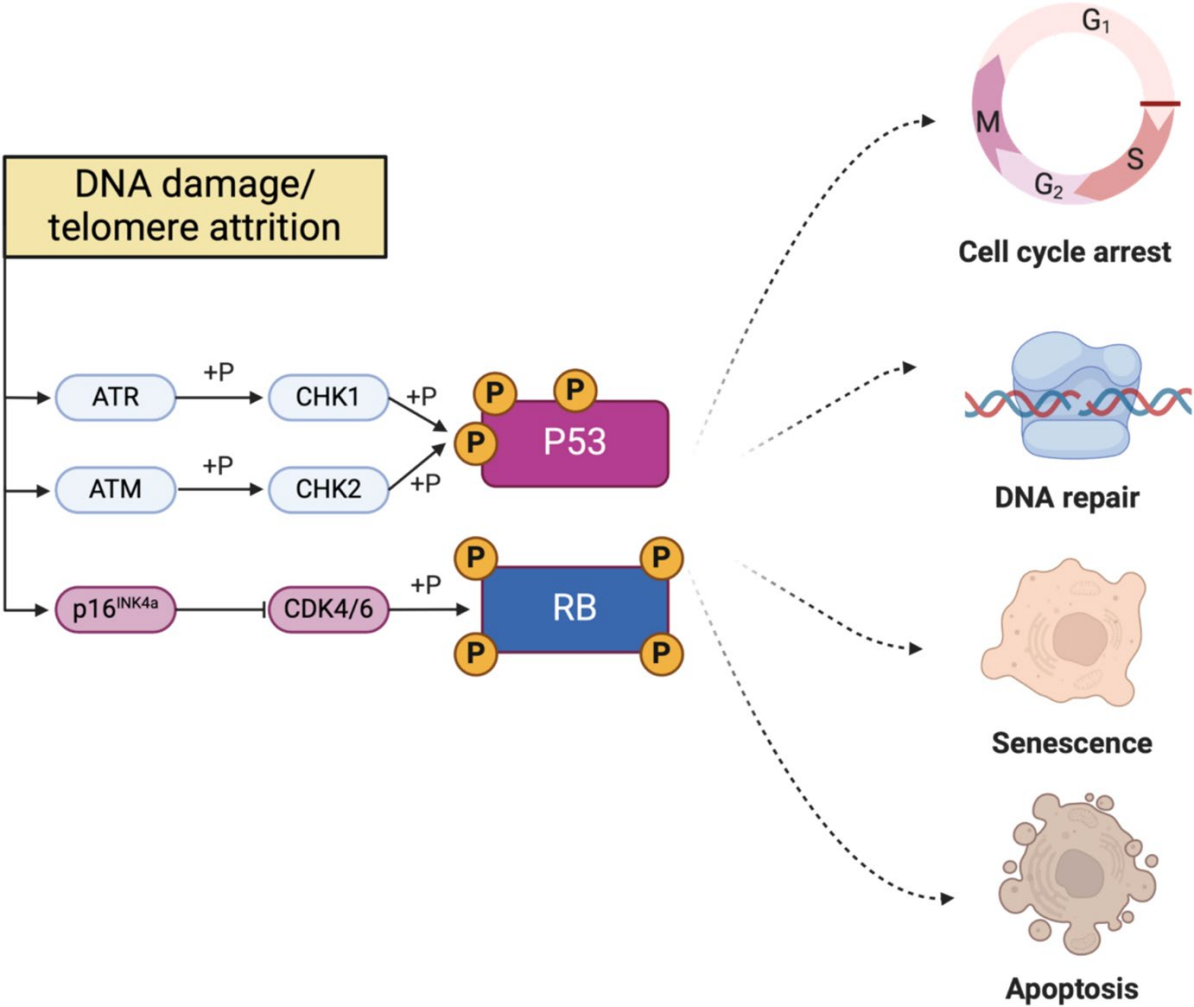
DNA damage and telomere attrition induced senescence

ATM and ATR also regulate checkpoint proteins CHK1 and CHK2, ensuring cells halt the cycle to repair damage or, if repair is unsuccessful, proceed to senescence or apoptosis (Bartek et al. 2007; Shiloh and Ziv 2013). Persistent DDR activation, as seen in aging cells with unresolved damage, stabilizes p53, leading to the transcription of p21, which inhibits cyclin-dependent kinases (CDKs) such as CDK2 and CDK4, effectively halting the cell cycle at the G1/S transition (Chen et al. 2006; Engeland 2022). Concurrently, the activation of the p16/Rb pathway reinforces this arrest by preventing E2F-mediated transcription necessary for S-phase entry, creating a robust blockade against cell cycle progression (Baker et al. 2016; Prieur et al. 2011). This dual-layered mechanism is essential for enforcing the senescence phenotype and preventing the propagation of damaged cells, although not every senescent cell type exhibits both (Fig. 2) (Herbig et al. 2004; Serrano et al. 1997).

Schematic representation of the DNA damage and telomere attrition response pathways. DNA damage activates ATM/ATR kinases, which phosphorylate CHK1/CHK2. Subsequently, CHK1/CHK2 phosphorylate p53, disrupting its interaction with the ubiquitin ligase MDM2 and preventing its ubiquitination and subsequent proteasomal degradation. Concurrently, p16^INK4a^ inhibits CDK4/6, leading to RB phosphorylation. These signaling cascades result in various cellular outcomes, including cell cycle arrest, DNA repair, senescence, or apoptosis, depending on the extent of damage and cellular context. Created with BioRender.com.

Beyond its role in general cellular aging, DDR-induced senescence is particularly relevant in the vascular system. Endothelial cells (ECs) exposed to chronic hemodynamic stress frequently exhibit accelerated telomere shortening. This process leads to the loss of telomeric integrity, triggering persistent DDR activation that enforces cellular senescence (Dominic et al. 2020; Warboys et al. 2014). Studies have demonstrated that ECs isolated from older adults exhibit critically short telomeres and elevated senescence markers, including upregulated p21 and p16 expression, which are pivotal for cell-cycle arrest (Dominic et al. 2020; Rossman et al. 2017). These senescent cells display impaired nitric oxide (NO) bioavailability due to reduced endothelial nitric oxide synthase (eNOS) activity. NO plays a fundamental role in maintaining vascular homeostasis by promoting vasodilation, inhibiting platelet aggregation, and reducing leukocyte adhesion to the endothelium.

In senescent ECs, reduced eNOS activity not only limits NO availability but also promotes oxidative stress through increased uncoupling of eNOS, leading to the production of reactive oxygen species (ROS) instead of NO (Hayashi et al. 2008). This dual impairment contributes to endothelial dysfunction, exacerbating vascular inflammation, stiffness, and the progression of atherosclerosis (Förstermann and Sessa 2012; Janaszak-Jasiecka et al. 2023).

### Mitochondrial dysfunction, oxidative stress and inflammatory crosstalk

**Fig. 3:**
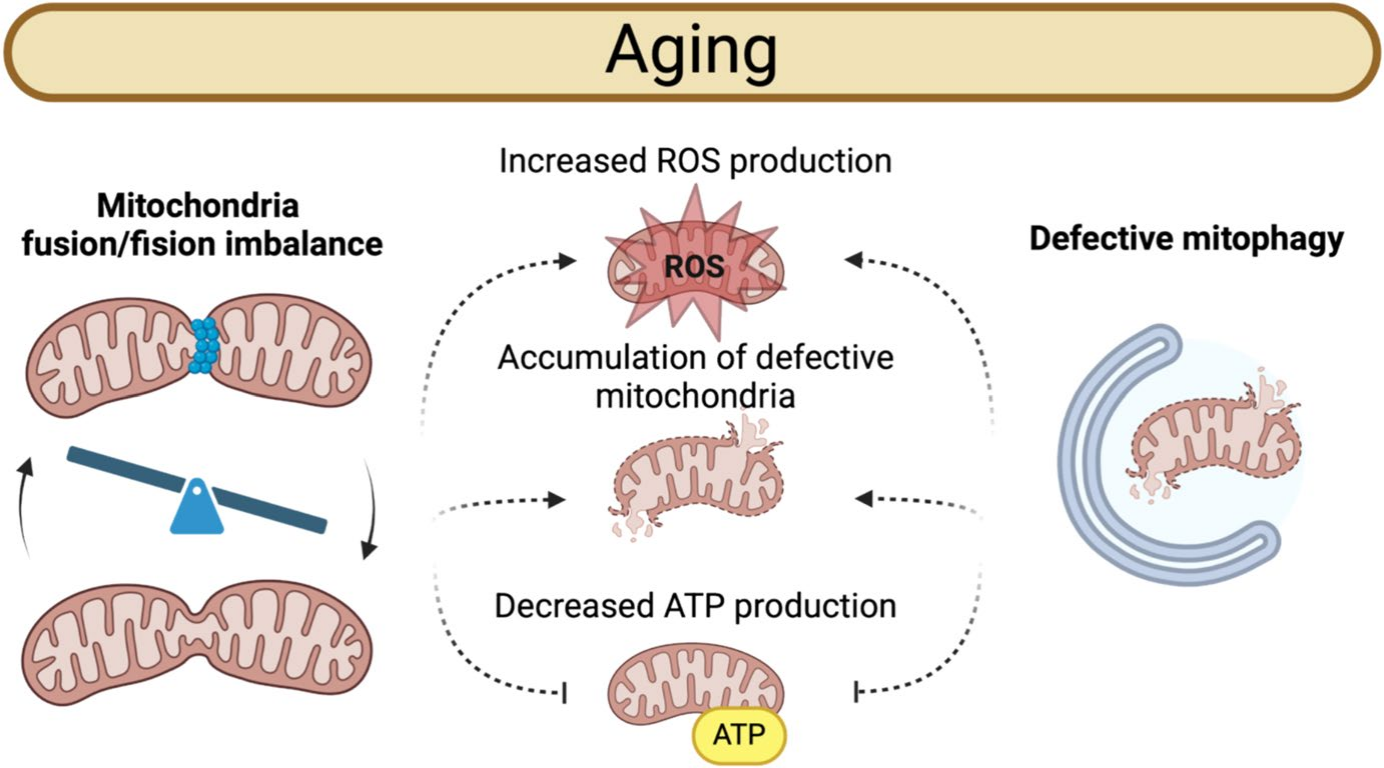
Mitochondrial impaired dynamics in Aging

Oxidative stress plays a pivotal role in driving cellular senescence, particularly through mitochondrial dysfunction, which arises from both intrinsic and extrinsic stressors during aging. Mitochondria experience a decline in membrane potential, impaired oxidative phosphorylation, and reduced ATP production, compromising their ability to sustain cellular energy demands (Finkel and Holbrook 2000; López-Otín et al. 2023; Miwa et al. 2022; Ni et al. 2015). A critical feature of mitochondrial dysfunction in aging is the imbalance between fusion and fission processes. Fusion, orchestrated by proteins such as mitofusin (MFN1/2) and optic atrophy 1 (OPA1), maintains mitochondrial integrity by mixing mitochondrial contents and diluting damaged components. Conversely, fission, regulated by dynamin-related protein 1 (DRP1), allows segregation and removal of damaged mitochondria via mitophagy. Aging disrupts this balance, with reduced expression of fusion-promoting proteins and overactivation of DRP1, leading to mitochondrial fragmentation and accumulation of defective organelles (Fig. 3) (Ashrafi and Schwarz 2013; Ni et al. 2015).

Aging disrupts mitochondrial homeostasis by causing an imbalance in fusion and fission dynamics, along with defects in mitophagy. These primary dysfunctions lead to increased reactive oxygen species (ROS) production, the accumulation of damaged mitochondria, and decreased ATP production. As a result, cellular function deteriorates, contributing to aging-related decline and senescence. Created with BioRender.com.

These dysfunctional mitochondria become major sources of ROS, which directly damage lipids, proteins, and DNA. Impaired mitophagy exacerbates this issue by failing to clear defective mitochondria, resulting in elevated ROS production. This oxidative stress activates redox-sensitive signaling pathways such as NF-κB and p38 MAPK, promoting the expression of inflammatory cytokines, including IL-6 and TNF-α, which reinforce SASP (Ashrafi and Schwarz 2013; Caja and Enríquez 2017; Chen et al. 2020).

Beyond impaired energy metabolism and increased ROS, dysfunctional mitochondria promote the release of mitochondrial DNA (mtDNA) and other danger-associated molecular patterns (DAMPs), which can activate the NLRP3 inflammasome and reinforce the SASP through NF-κB signaling, amplifying local and systemic inflammation (Acosta et al. 2013; Chen et al. 2020; Ya and Bayraktutan 2024).

In aging vascular cells, defective mitophagy fails to eliminate damaged mitochondria, perpetuating ROS production and redox-sensitive senescence signaling. This is particularly relevant in endothelial cells, where impaired mitophagy has been linked to the loss of mitochondrial quality control proteins such as PINK1 and Parkin (Chen et al. 2020; Miwa et al. 2022).

Additionally, energy sensors such as AMPK and nutrient-sensing pathways like mTOR orchestrate the balance between mitochondrial health and cellular stress responses. Inhibition of AMPK or overactivation of mTOR has been associated with mitochondrial dysfunction and endothelial senescence, linking metabolic cues to aging-related vascular decline (Caja and Enríquez 2017; Ya and Bayraktutan 2024).

In ECs, mitochondrial dysfunction disrupts endothelial nitric oxide synthase (eNOS) activity, reducing nitric oxide (NO) bioavailability (Addabbo et al. 2009; Caja and Enríquez 2017). Additionally, the loss of mitochondrial quality control proteins, such as PGC-1α, perpetuates the accumulation of dysfunctional mitochondria, further aggravating endothelial dysfunction (Kant et al. 2022; Valle et al. 2005).

Similarly, in vascular smooth muscle cells (VSMCs), ROS are key drivers of senescence, particularly through oxidative stress and DNA damage. Senescent VSMCs play a critical role in vascular aging by compromising the structural integrity of blood vessels. VSMCs are responsible for maintaining vascular tone and extracellular matrix stability (Scotti et al. 2024). The accumulation of senescent VSMCs exacerbates atherosclerotic plaque instability, increasing the risk of rupture and thrombosis (Gorenne et al. 2006; Grootaert et al. 2018; Li et al. 2024).

### Senescence-associated secretory phenotype (SASP)

SASP is characterized by the release of cytokines (e.g., IL-6, IL-8), chemokines, growth factors, and proteases (Birch and Gil 2020; Evans et al. 2023). It has both physiological and pathological roles, mediating various aspects of cellular and tissue homeostasis.

Physiologically, SASP can serve as a signal for immune clearance of senescent cells, aiding in tissue repair and limiting the accumulation of potentially oncogenic cells. Components such as IL-6 and IL-8 recruit immune cells to the site of damage, facilitating the removal of senescent cells (Acosta et al. 2013; Kale et al. 2020). In chronic conditions, SASP promotes tissue dysfunction through a variety of interconnected mechanisms. SASP fosters smooth muscle proliferation, extracellular matrix remodeling, and endothelial barrier dysfunction, accelerating atherosclerotic plaque formation (Freund et al. 2010; Gardner et al. 2015; Khosla et al. 2020). Factors such as vascular endothelial growth factor (VEGF) induce aberrant angiogenesis by promoting excessive blood vessel formation, often in a disorganized manner. Meanwhile, MMPs degrade extracellular matrix components, disrupting the structural support of blood vessels and weakening vascular integrity. This imbalance between angiogenesis and matrix degradation exacerbates vascular dysfunction, contributing to age-related vascular diseases such as atherosclerosis and hypertension (Carmeliet and Jain 2011; Kessenbrock et al. 2010). Additionally, in VSMC, SASP drives calcification, further reducing arterial flexibility (Burton et al. 2010; Zuccolo et al. 2020).

Pro-inflammatory cytokines like IL-6 and IL-8 amplify the senescent cell burden by inducing paracrine senescence in neighboring cells. These cytokines trigger downstream signaling pathways, such as JAK/STAT and MAPK, in adjacent vascular cells, reinforcing senescence and promoting the SASP cascade. This mechanism not only increases local inflammation but also spreads senescence-associated phenotypes across the vascular tissue, accelerating dysfunction and remodeling (Acosta et al. 2013; Coppé et al. 2010; Freund et al. 2010).

In a similar fashion to ECs, senescent VSMCs experience phenotypic switching triggered by oxidative stress, DNA damage, and inflammation, leading to reduced contractility and heightened SASP. These cells also produce matrix metalloproteinases (MMPs), degrading the extracellular matrix and promoting vascular stiffness (Childs et al. 2017; Gardner et al. 2015; Grootaert et al. 2018).

SASP components also activate redox-sensitive transcription factors, including NF-κB, which plays a central role in chronic inflammation and oxidative stress during aging. NF-κB activation is triggered by persistent ROS production and cytokine signaling, leading to the upregulation of pro-inflammatory mediators like IL-6, TNF-α, and MCP-1. These factors amplify inflammatory pathways, creating a self-reinforcing cycle of oxidative stress and tissue damage that contributes to vascular dysfunction and aging-related pathologies (Hayden and Ghosh 2014; Salminen et al. 2008; Ya and Bayraktutan 2024). The SASP exemplifies a vicious circle, acting not only as a consequence of senescence but also as a potent driver that perpetuates further senescence and promotes cancer development (Takasugi et al. 2023).

### Epigenetic and metabolic factors

Epigenetic modifications play a critical role in regulating vascular senescence and its downstream effects. Changes such as DNA methylation, histone modifications, and chromatin remodeling contribute to altered gene expression patterns associated with aging. For example, hypermethylation of promoters for DNA repair genes may exacerbate genomic instability, while hypomethylation of inflammatory genes can amplify SASP-related signaling (López-Otín et al. 2023; Sen et al. 2016). Histone acetylation, regulated by enzymes like histone acetyltransferases (HATs) and deacetylases (HDACs), is another pivotal mechanism that influences chromatin accessibility and transcriptional activity, thereby affecting pathways such as p53/p21 and NF-κB activation (Hayakawa et al. 2015; Sen et al. 2016; Yao and Rahman 2012).

Metabolic factors also interconnect with epigenetic mechanisms to drive senescence. Mitochondrial dysfunction and reduced NAD + levels impair the activity of sirtuins, a family of NAD + -dependent deacetylases. This disruption further affects the regulation of histone modifications and metabolic pathways. This decline in sirtuin activity exacerbates oxidative stress, inflammation, and mitochondrial fragmentation, all hallmarks of vascular aging. Moreover, the reprogramming of glucose and lipid metabolism in senescent cells shifts energy production toward glycolysis and fatty acid synthesis, fueling the secretion of SASP factors. Together, these epigenetic and metabolic changes create a feedback loop that accelerates senescence and contributes to the development of cardiovascular diseases such as atherosclerosis and hypertension (Banerjee et al. 2022; Evangelou et al. 2022; Katsuumi et al. 2018).

## Clinical implications of vascular senescence

### Hypertension and arterial stiffness

Arterial stiffness is defined as the reduced ability of the arteries to expand and contract in response to pressure changes. It is a hallmark of vascular aging and is strongly associated with isolated systolic hypertension. The loss of arterial elasticity is primarily driven by structural changes in the vascular wall, including the accumulation of senescent VSMCs and increased deposition of extracellular matrix proteins like collagen, coupled with a reduction in elastin (Li et al. 2024; Martínez-Revelles et al. 2017). Senescent VSMCs exhibit altered phenotypes characterized by reduced contractility and the secretion of MMPs, which degrade elastin and disrupt collagen crosslinks, weakening arterial compliance (Mitchell et al. 2010).

Additionally, senescent ECs exhibit reduced vasodilatory capacity and increased vasoconstrictive tendencies, partly due to impaired eNOS activity, as discussed in “DNA Damage and Telomere Attrition” section. This imbalance shifts vascular tone toward heightened resistance, contributing to increased systemic vascular resistance. These changes result in thicker and less compliant arteries, leading to elevated systolic blood pressure (van der Feen et al. 2020; Förstermann and Sessa 2012; Janaszak-Jasiecka et al. 2023).

Mitochondrial dysfunction plays a central role in these processes by reducing nitric oxide availability, increasing vascular oxidative stress, and sustaining pro-inflammatory signaling, thereby contributing to the pathogenesis of hypertension and atherosclerosis (Chen et al. 2020; Miwa et al. 2022; Ya and Bayraktutan 2024).

With aging, arterial stiffness contributes to a range of cardiovascular complications. The reduced ability of arteries to buffer pulsatile blood flow increases left ventricular afterload, which can lead to left ventricular hypertrophy and heart failure. Additionally, the stiffened arteries impair coronary perfusion during diastole, exacerbating myocardial ischemia. This pathophysiological process is further compounded by the secretion of pro-inflammatory and pro-fibrotic factors from senescent cells, which perpetuate vascular remodeling and systemic inflammation (Gevaert et al. 2017; Salman et al. 2024).

### Atherosclerosis and plaque vulnerability

While hypertension and arterial stiffness primarily affect vascular tone and pressure regulation, cellular senescence also contributes to the structural changes that underlie atherosclerosis. The accumulation of senescent cells in the intima and media layers of arteries plays a critical role in both the initiation and progression of atherosclerotic plaques.

Within the intima, senescent ECs contribute to increased permeability and leukocyte adhesion, fostering an inflammatory environment that promotes plaque formation (Bloom et al. 2023; Bloom et al. 2022).

While senescent ECs impact the vascular lumen and initiate inflammation, VSMCs located in the media layer contribute to structural destabilization of the arterial wall through degradation of collagen and elastin, promoting plaque rupture, which may precipitate infarction or stroke (Evangelou et al. 2022; Gevaert et al. 2017).

**Fig. 4:**
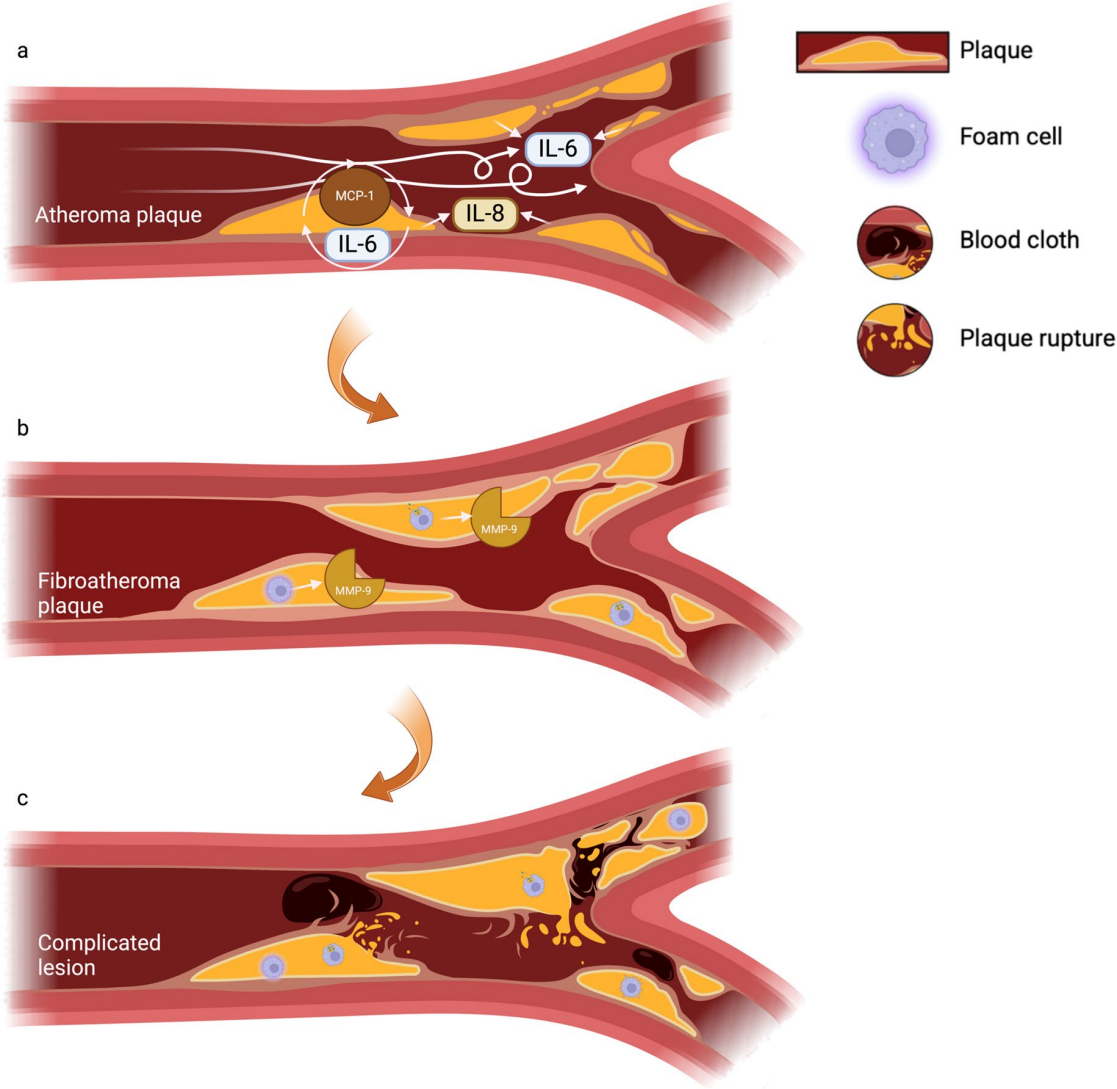
Progression of atherosclerosis and its connection to vascular senescence

In addition to vascular cells, immune cells such as macrophages within atherosclerotic plaques can also acquire senescent features, characterized by a pro-inflammatory phenotype and the secretion of SASP factors such as IL-6 and TNF-α. These inflammatory mediators amplify local tissue damage, recruit additional immune cells, and exacerbate the inflammatory microenvironment (Childs et al. 2016). Furthermore, the SASP from senescent immune cells also enhances proteolytic activity through MMP-9 secretion, further increasing the risk of plaque rupture (Vellasamy et al. 2022), as shown in Fig. 4.

The image illustrates key stages in atherosclerosis development, highlighting the role of senescent cells in endothelial dysfunction and plaque formation. (a) In the early stages, pro-inflammatory factors such as IL-6 and IL-8, released by dysfunctional endothelial cells and senescent cells, promote monocyte adhesion and migration into the vascular endothelium, facilitated by chemokines like MCP-1. These factors reinforce senescence and generate low-grade inflammatory background systemically. (b) Monocytes differentiate into macrophages, engulf oxidized lipids, and form foam cells, contributing to plaque expansion. Senescent vascular smooth muscle cells and foam cells secrete matrix metalloproteinases that degrade collagen and elastin. (c) In advanced stages, plaque instability, driven by matrix metalloproteinase activity and the persistent presence of senescent cells, can lead to plaque rupture and thrombus formation, increasing the risk of cardiovascular events such as myocardial infarction or stroke. Created with BioRender.com.

The accumulation of senescent cells within plaques also drives calcification processes by promoting osteogenic differentiation within VSMCs. In the context of senescence, VSMCs exhibit a phenotypic switch, expressing markers such as alkaline phosphatase and osteopontin, which are typically associated with bone-forming cells. This osteogenic transformation is driven by chronic inflammation and oxidative stress (Liu et al. 2013; Nakano-Kurimoto et al. 2009; Zuccolo et al. 2020).

SASP factors like IL-6 and TNF-α mediate this process by activating signaling pathways such as BMP (bone morphogenetic protein) and Wnt/β-catenin, which are critical regulators of calcification. These molecular changes not only drive the deposition of hydroxyapatite crystals in the vascular wall but also stiffen the vessel, impairing its ability to adapt to hemodynamic changes (Burton et al. 2010; Childs et al. 2017).

Additionally, senescent ECs contribute to this pathological calcification by altering the local microenvironment. They secrete extracellular vesicles rich in calcium and phosphate, which act as nucleation sites for crystal formation. These vesicles, combined with the reduced expression of calcification inhibitors like matrix Gla-protein (MGP), further enhance the calcification process (Mas-Bargues et al. 2022). This combination of structural weakening and calcification underscores the pathological role of senescence in cardiovascular disease progression and highlights the therapeutic potential of targeting senescent cells to stabilize plaques and prevent acute cardiovascular events (Mas-Bargues et al. 2022; Vellasamy et al. 2022; Xiang et al. 2022).

### Associated age-related disorders

Beyond hypertension and atherosclerosis, vascular senescence contributes to a range of complications across different organ systems, further exacerbating age-related disease.

Systemic low-grade inflammation (Inflammaging): The SASP factors secreted by senescent ECs not only exert local effects but also circulate systemically, driving widespread inflammation throughout the body. The pro-inflammatory cytokines, chemokines, and proteases secreted by these cells, including IL-6, IL-8, TNF-α, and MMPs, disseminate through the bloodstream, altering the function of distant tissues and organs. This systemic release of SASP factors leads to chronic low-grade inflammation, commonly referred to as “inflammaging”, which significantly contributes to the pathology of various age-related diseases, including diabetes, neurodegenerative disorders, and chronic kidney disease (Birch and Gil 2020; Dai et al. 2019; Katsuumi et al. 2018; Sahu et al. 2022; Tchkonia et al. 2013). Moreover, systemic SASP influences metabolic homeostasis by impairing insulin signaling pathways, contributing to insulin resistance and metabolic syndrome (Murakami et al. 2022; Shimi et al. 2024).

Neurovascular Dysfunction and Cognitive Decline: Vascular senescence is a key factor in small-vessel disease in the brain, characterized by reduced perfusion and loss of microvascular integrity. This condition increases the risk of cognitive decline, vascular dementia, and ischemic stroke. Senescent ECs within cerebral vessels impair blood–brain barrier (BBB) function and exacerbate neuroinflammation, further contributing to neuronal injury (Iadecola and Gottesman 2019; Wang et al. 2024).

Renal Dysfunction and Chronic Kidney Disease (CKD): Microvascular senescence in the kidneys impairs glomerular filtration and contributes to age-related kidney dysfunction, including CKD. Senescent renal ECs exhibit reduced nitric oxide (NO) bioavailability and heightened oxidative stress, leading to capillary rarefaction and impaired nephron perfusion. The SASP further amplifies renal fibrosis by promoting pro-inflammatory and pro-fibrotic signaling pathways (Dai et al. 2019; Docherty et al. 2019; Melk et al. 2009).

Peripheral Artery Disease (PAD): Senescence-induced endothelial dysfunction contributes to reduced circulation in the extremities, characteristic of PAD. The impaired angiogenic capacity of senescent endothelial and vascular smooth muscle cells limits collateral vessel formation, worsening ischemic conditions in affected tissues. This is accompanied by an inflammatory milieu driven by SASP factors, exacerbating tissue damage and delaying wound healing (Minami et al. 2019; Sosińska-Zawierucha et al. 2018), a hallmark of severe PAD.

The broad impact of SASP as a driver of multi-organ dysfunction in aging highlights the therapeutic potential of targeting systemic SASP to mitigate age-related diseases.

## Therapeutic strategies and interventions

### Senolytics and senomorphics

Senolytic drugs selectively induce apoptosis in senescent cells. These agents target pathways that are specifically upregulated in senescent cells, such as BCL-2 and p53 signaling, enabling selective elimination while sparing normal cells. Dasatinib and quercetin combination has demonstrated efficacy in reducing senescent cell populations in humans (Hickson et al. 2019; Nieto et al. 2024; Tkemaladze 2023) while navitoclax (ABT-263) has shown promising results improving arterial stiffening and reducing plaque formation in mice (Karnewar et al. 2024; Mahoney et al. 2021). Navitoclax is being studied in clinical trials for solid tumor treatment (Corcoran et al. 2024; Harrison et al. 2022). Dasatinib and quercetin combination or fisetin are currently being investigated in clinical trials (ClinicalTrials.gov identifiers: NCT05595499, NCT04733534, and NCT06113016) for their potential to target cellular senescence and modulate SASP in oncology patients.

Additionally, senolytics have shown promise in reducing systemic inflammation driven by SASP factors, which can have downstream benefits for age-related pathologies beyond the vasculature. By removing senescent cells, these drugs decrease the secretion of pro-inflammatory cytokines like IL-6 and TNF-α, mitigating the chronic low-grade inflammation associated with age (Herbstein et al. 2024; Tchkonia et al. 2013). Although senescent cells contribute to aging and chronic inflammation through the SASP, they also play positive roles in tissue repair, wound healing, and tumor suppression. For instance, senescent cells can secrete growth factors, such as platelet-derived growth factor (PDGF) and VEGF, which facilitate tissue regeneration and limit the proliferation of damaged cells. These secreted factors help recruit immune cells to sites of injury, promoting tissue remodeling and homeostasis (Demaria et al. 2014; Rhinn et al. 2019). Therefore, challenges remain in optimizing the specificity, dosing, and long-term safety of these therapies to minimize off-target effects and maintain immune surveillance mechanisms essential for tumor suppression and tissue repair (Khosla 2023; Kirkland and Tchkonia 2020).

Senomorphics (also called senostatics) modulate the SASP without eliminating senescent cells, aiming to alleviate the inflammatory burden. These agents, with rapamycin as the gold standard, work by inhibiting key signaling pathways involved in SASP production, such as the NF-κB, MAPK, and mTOR pathways (Aziz et al. 2024; Ya and Bayraktutan 2024). Other senomorphics, like JAK inhibitors, target cytokine signaling cascades to diminish the pro-inflammatory environment created by senescent cells (Valenzuela 2022; Xu et al. 2016; Xu et al. 2015). Among drugs with senomorphic properties, metformin is currently being studied in the Targeting Aging with Metformin (TAME) trial, which aims to assess its potential to delay the onset of age-related diseases in older adults (Abdelgawad et al. 2023; Barzilai et al. 2016).

Clinical trials exploring these agents are ongoing, with promising results indicating reduced systemic inflammation and improved physical function in older adults. However, questions remain regarding the long-term safety and potential off-target effects, such as impairing immune responses or altering the microenvironment necessary for normal tissue homeostasis. Further research is necessary to optimize senotherapies and ensure their safe and effective integration into clinical practice.

### Anti-inflammatory and antioxidant approaches

Since chronic inflammation and oxidative stress are key drivers of vascular senescence, anti-inflammatory agents and antioxidants have been proposed to slow endothelial aging. These compounds aim to mitigate the pro-inflammatory microenvironment and reduce oxidative damage, thereby preserving vascular integrity. For instance, colchicine, traditionally used for gout, has demonstrated potential in reducing vascular inflammation and the progression of atherosclerosis by inhibiting the NLRP3 inflammasome pathway (Libby 2021). Similarly, IL-1β inhibitors like canakinumab have demonstrated efficacy in lowering systemic inflammation markers, such as C-reactive protein (CRP), which are closely associated with vascular aging and cardiovascular events (Ridker et al. 2017).

Antioxidants, such as N-acetylcysteine, target ROS and improve redox balance in vascular cells. By replenishing intracellular glutathione levels, N-acetylcysteine can reduce oxidative stress and prevent ROS-induced endothelial dysfunction (Sun 2010). Recent studies, such as those investigating the GlyNAC (glycine and N-acetylcysteine) supplementation, have demonstrated promising effects in improving mitochondrial function, reducing oxidative stress, and enhancing physical performance in aging populations. This combination restores glutathione levels and corrects nutrient deficiencies associated with aging, reinforcing its potential as a therapeutic intervention to mitigate vascular senescence (Kumar et al. 2023; Lizzo et al. 2022).

### Lifestyle interventions

Lifestyle factors play a crucial role in vascular aging, influencing endothelial function, inflammation, and oxidative stress: Among these factors, exercise and diet stand out as key modulators of vascular health.

#### Exercise

Regular aerobic training improves endothelial function by enhancing NO bioavailability and reducing oxidative stress. It also promotes mitochondrial efficiency and biogenesis, delaying cellular senescence. Resistance training has been shown to improve vascular stiffness and reduce arterial aging markers, making exercise a multifaceted approach to combating vascular senescence (Meng et al. 2023; Rossman et al. 2017).

#### Diet

A diet rich in polyphenols, such as the Mediterranean-style diet, is associated with lower systemic inflammation and reduced telomere attrition. Diets high in omega-3 fatty acids have demonstrated benefits in reducing vascular inflammation and improving endothelial function (Delgado-Lista et al. 2022; Estruch et al. 2018). A diet low in saturated fat reduces ApoB-containing cholesterol particles, thereby reducing the risk of atherosclerosis and vascular senescence associated with elevated lipid levels. Studies have demonstrated that such dietary interventions improve lipid profiles by decreasing LDL cholesterol and ApoB concentrations, which are critical contributors to plaque formation and vascular inflammation. Additionally, reducing saturated fat intake modulates systemic inflammation and oxidative stress, further mitigating the pathways leading to vascular aging (Hooper et al. 2020; Perna and Hewlings 2023).

#### Alcohol and tobacco

Consumption of tobacco and alcohol significantly accelerates vascular senescence. Tobacco smoke contains numerous oxidants and pro-inflammatory agents, including nicotine, that induce oxidative stress and cellular senescence in both ECs and VSMC (Centner et al. 2020; Csiszar et al. 2009). Similarly, alcohol has been associated with increased arterial stiffness, potentially due to its impact on lipid metabolism, oxidative stress, and inflammatory processes (Vallée 2023).

#### Sleep

Sleep deprivation has been identified as a significant risk factor for nearly every major disease, including cardiovascular disorders (Liew and Aung 2021). Unsurprisingly, sleep apnea -a common sleep disorder -has been strongly associated with increased arterial stiffness and accelerated vascular aging (Lisan et al. 2021). Ensuring adequate, high-quality sleep is essential for maintaining vascular health and promoting overall well-being.

### Gene therapy and advanced modalities

Future interventions may include advanced gene-editing strategies, such as CRISPR-Cas9 and base editing, to restore telomere length or suppress key senescence pathways like p16 and p21. CRISPR-Cas9 can precisely target and modify senescence-associated genes, while base editing enables single-nucleotide changes, offering a more refined approach to genetic correction. A genome-wide CRISPR-Cas9 screening conducted in 2021 identified KAT7 as a key gene promoting cellular senescence. Ablation of KAT7 alleviated cellular senescence and organ aging, enhancing both health span and life span in aged mice (Wang et al. 2021). These techniques aim to reverse cellular aging, improve vascular function, and prevent age-related pathologies.

RNA-based therapies targeting pro-senescence genes, including siRNAs and mRNAs, are also under investigation. These therapies aim to silence key drivers of senescence, such as p16 and p21, to prevent the progression of cellular aging. For example, siRNAs targeting p16 have demonstrated significant potential in reducing senescent cell burden and SASP secretion in preclinical models, leading to improved tissue homeostasis and reduced inflammation (Buj et al. 2021). Additionally, mRNA-based interventions are being developed to enhance the expression of protective genes, such as telomerase reverse transcriptase (TERT), which can restore telomere length and delay the onset of senescence. For instance, preclinical studies have shown that TERT mRNA therapies can rejuvenate cellular functions, improve telomeric integrity, and reduce markers of aging-related stress in vascular cells (Bernardes de Jesus et al. 2012; Mojiri et al. 2021).

Exosome-based delivery systems are being investigated as a novel approach for transporting therapeutic RNAs or proteins directly to vascular cells. Exosomes are naturally occurring vesicles capable of delivering a variety of cargo, including nucleic acids, proteins, and lipids, to target cells. This unique property makes them attractive candidates for precision medicine in vascular therapy. For instance, exosomes loaded with different cargos demonstrated efficacy in reducing senescent cell burden and improving vascular function (Luan et al. 2017; Tariq et al. 2025). Recent advancements in surface modification techniques, such as ligand-receptor targeting and functionalized coatings, have enabled the precise delivery of exosomes to specific vascular tissues. These modifications enhance therapeutic efficacy while minimizing systemic side effects, making exosome-based therapies a promising avenue for clinical applications in vascular health Challenges remain, however, in scaling up exosome production and ensuring their stability during storage and administration, but ongoing research continues to refine these technologies, paving the way for their clinical application (Kalluri and LeBleu 2020; Luan et al. 2017).

Lastly, D-galactose-formulated nanoparticles exploit the increased β-galactosidase activity in senescent cells to deliver pharmacological agents. These agents remain inactive until the galactose coating is metabolized by the target cells. This strategy aims to enhance selectivity and reduce the risk profile associated with senotherapies (González-Gualda et al. 2020; Wei et al. 2023).

## Future directions

Despite growing insights, multiple unanswered questions and challenges remain in the diagnosis and development of therapies for senescence. Addressing these challenges is critical to advancing the field of vascular senescence and its therapeutic implications. For example, developing reliable biomarkers for the early detection of vascular senescence remains a key priority. Biomarkers such as circulating extracellular vesicles carrying senescence-associated proteins or microRNAs may provide non-invasive tools for monitoring senescence progression in vivo (Fielding et al. 2024; Fielding et al. 2022; Salman et al. 2024; Sorokina et al. 2023).

Similarly, optimizing delivery mechanisms for therapies such as senolytics, senomorphics, and RNA-based approaches is essential to enhance specificity and minimize off-target effects, particularly in older populations with comorbidities. Advances in nanotechnology, such as lipid nanoparticles and polymeric micelles, have shown promise in improving drug delivery efficiency by targeting specific cell types and reducing systemic exposure. For instance, lipid nanoparticles have been successfully used in mRNA-based COVID-19 vaccines, demonstrating their potential for safe and effective delivery of nucleic acids (Baden et al. 2021; Polack et al. 2020).

Personalized medicine approaches that integrate biomarkers and senescence profiling could enable tailored interventions, optimizing efficacy while reducing adverse effects. Moreover, leveraging multi-omics technologies, including genomics, proteomics, and metabolomics, to map the senescent phenotype could identify novel targets and enhance the understanding of the molecular underpinnings of vascular aging (Shen et al. 2024; Song et al. 2022).

Artificial intelligence and machine learning have the potential to analyze large-scale omics data and predicting individualized responses to therapies, further advancing medicine strategies for combating chronic diseases and vascular aging (Bernard et al. 2023; He et al. 2023). LLMs, and more recently, reasoning models, are transforming medicine and drug discovery by enabling advanced data analysis and facilitating innovative research approaches. Those models contribute to target identification, clinical trial design, regulatory decision-making, and pharmacovigilance, expediting the development of new treatments. Integrating LLMs into personalized medicine holds promise for tailoring treatments to individual patient profiles, potentially improving outcomes and reducing adverse effects. As these models evolve, their applications are expected to expand, further revolutionizing medical research and patient care (Bhattacharya et al. 2024; Chakraborty et al. 2023).

## Conclusions

Vascular senescence stands at the intersection of cellular aging and cardiovascular disease. Its hallmark features—telomere attrition, oxidative stress, and the pro-inflammatory SASP—collaborate to drive endothelial dysfunction, arterial stiffness, and atherogenesis. Evidence suggests that interventions targeting senescent cells, whether through pharmacological senolytics, anti-inflammatory regimens, or lifestyle modifications, offer a promising avenue for preventing or slowing the progression of age-related vascular diseases. However, further research is needed to define optimal treatment regimens, evaluate long-term safety, and develop precise diagnostic tools. The integration of basic science discoveries with clinical insights and AI, may help mitigate the growing burden of cardiovascular disease in aging populations.

## Data Availability

No datasets were generated or analysed during the current study.
